# An investigation of genetic polymorphisms in heparan sulfate proteoglycan core proteins and key modification enzymes in an Australian Caucasian multiple sclerosis population

**DOI:** 10.1186/s40246-020-00264-6

**Published:** 2020-05-12

**Authors:** Rachel K. Okolicsanyi, Julia Bluhm, Cassandra Miller, Lyn R. Griffiths, Larisa M. Haupt

**Affiliations:** Genomics Research Centre, Institute for Health and Biomedical Innovation, School of Biomedical Sciences, Queensland University of Technology, Musk Avenue, Kelvin Grove, Brisbane, Queensland 4059 Australia

**Keywords:** Syndecans, Glypicans, HSPG, Multiple sclerosis, SNP, EXT1, SULF1

## Abstract

Multiple sclerosis (MS) is a chronic inflammatory demyelinating disease affecting the central nervous system in young adults. Heparan sulfate proteoglycans (HSPGs) are ubiquitous to the cell surface and the extracellular matrix. HSPG biosynthesis is a complex process involving enzymatic attachment of heparan sulfate (HS) chains to a core protein. HS side chains mediate specific ligand and growth factor interactions directing cellular processes including cell adhesion, migration and differentiation. Two main families of HSPGs exist, the syndecans (SDC1-4) and glypicans (GPC1-6). The SDCs are transmembrane proteins, while the GPC family are GPI linked to the cell surface. SDC1 has well-documented interactions with numerous signalling pathways. Genome-wide association studies (GWAS) have identified regions of the genome associated with MS including a region on chromosome 13 containing GPC5 and GPC6. International studies have revealed significant associations between this region and disease development. The exostosin-1 (EXT1) and sulfatase-1 (SULF1) are key enzymes contributing to the generation of HS chains. EXT1, with documented tumour suppressor properties, is involved in the initiation and polymerisation of the growing HS chain. SULF1 removes 6-*O*-sulfate groups from HS chains, affecting protein-ligand interactions and subsequent downstream signalling with HS modification potentially having significant effects on MS progression. In this study, we identified significant associations between single nucleotide polymorphisms in SDC1, GPC5 and GPC6 and MS in an Australian Caucasian case-control population. Further significant associations in these genes were identified when the population was stratified by sex and disease subtype. No association was found for EXT1 or SULF1.

## Introduction

Multiple sclerosis (MS) is one of the most common neurological diseases affecting young adults in Western society [1]. It is an inflammatory, demyelinating, neurodegenerative disease affecting the central nervous system (CNS) destroying myelin, oligodendrocytes, axons and neurons [2, 3]. This disease is predominantly found in Caucasians with an onset between 18 and 40 years [4]. Onset of MS varies from early childhood to adult life, affecting more than 25,000 people in Australia in 2017 [5] and 2.3 million people worldwide in 2013 [6]. Symptoms include weakness in one or more limbs, visual disturbances and balance problems. As MS progresses, fatigue, bladder and bowel dysfunction, numbness, tremor, spasticity and/or depression may occur [7]. Individuals of Northern European ancestry, including those from Scandinavia, Iceland, the British Isles and North America, exhibit increased risk compared with African Americans (0.005%) [8, 9]. There is a female predominance of about 3:1 [10].

MS is a complex genetic disease characterised by polygenic heritable components and multifaceted gene-environment interactions and factors [11]. Several studies have investigated the interaction between genes and environmental factors [12] with underlying genetic factors implicated in determining familial clustering and individual susceptibility [13]. MS can be categorised into four subtypes: relapsing remitting MS (55%; RRMS); secondary progressive MS (30%; SPMS); primary progressive MS (10%; PPMS) and progressive relapsing MS (5%; PRMS) [9, 14]. The severity and frequency of attacks as well as the reversibility of damage suffered during these attacks varies with disease state [9, 14]. Females are twice as likely to develop RRMS as males [14, 15] and due to the progressive axonal loss, 50% of RRMS cases convert to the late phase SPMS within 8 to 20 years [9]. The conversion to more severe forms of disease results in progressive disability and increasing irreversible damage with fewer remitting stages and no symptomatic relief [7, 9, 13, 16–18].

Neurodegeneration is the major cause of permanent neurological disability in MS patients [13]. In the CNS, neurons are composed of an oligodendrocyte axon surrounded by myelin, a lipid-rich insulating sheath [19] that allows rapid conduction of nerve impulses in the form of an action potential [4]. Degradation of the myelin sheath, and hence axonal damage, resulting from deregulation of the immune system results in partial or complete blockage of CNS signal pathways [9, 13]. The formation of scar tissue (“sclerosis” or demyelinated plaques) in multiple locations within the CNS [9, 13] produces symptoms common to MS including sensory disturbances [9, 16]. The mechanisms by which damage occurs in different subtypes is thought to differ; however, the outcome remains the same—primary demyelination and secondary axonal degeneration [14].

Early genetic studies associated the major histone compatibility (MHC) complex with MS and identified alleles of the human leukocyte antigen (HLA) region on chromosome 6q21 (6q21-23) common to MS [20]. Between 15 and 60% of the genetic aetiology of MS has been attributed to this region [21]. A more recent strategy employed to identify genetic markers of MS is genome-wide association studies (GWAS). These studies examine single nucleotide polymorphisms (SNPs) across the genome and provide additional genetic information about common complex disorders including identification of polymorphisms associated with diseases such as cancer and MS ([22], 2011). In 2008, a GWAS identified non-HLA genes involved in MS [11]. Most of the genes identified were in immunological pathways (eg. interleukin-7 receptor [11, 23–27]; with confirmation of their impact gained through gene expression studies.

To date, the complex processes and factors that lead to demyelination, axonal damage, progressive neurological symptoms and the loss of immune homeostasis remain poorly understood. However, during nervous system development, the growing axons are thought to be guided towards their target by a complex that may include heparan sulfate proteoglycans (HSPGs) [28].

HSPGs are found in both vertebrates and invertebrates [29, 30] and are ubiquitous to the cell surface and the extracellular matrix (ECM). HSPGs belong to a large family of proteoglycans (PG) that are composed of one or more variably sulfated glycosaminoglycan chains (GAGs) attached to a core protein. Heparan sulfate (HS) chains undergo a complex, highly coordinated, sequential biosynthesis process resulting in a disaccharide chain consisting of *N-* and *O*-sulfated residues (Habuchi, [31]). This process involves a number of enzymes responsible for the initiation and subsequent modification of the growing GAG chain, resulting in a chain of variable length and sulfation pattern (Habuchi, [31]). Two of these enzymes are exostosin-1 (EXT1) and sulfatase-1 (SULF1). EXT1 encodes a glycotransferase protein that catalyses polymerisation of the GAG chain [32, 33]. Complete abrogation of EXT1 results in embryonic lethality during gastrulation due to the absence of heparan sulfate [34, 35].

HSPGs interact and bind a variety of growth factors (eg. FGF-2, TGF-β), chemokines and morphogenes [36] to mediate downstream signalling and subsequent cellular processes. The ligand-binding properties of HS are associated with chain length (initiated by EXT1 and others), but more particularly, specific sulfation patterns conferred through the action of enzymes such as SULF1. SULF1 removes 6-*O*-sulfate groups, altering binding sites for signalling molecules (eg. FGF2) [37] resulting in altered cell function. Mutations of SULF1 can result in a loss of function, potentially leading to lethality [38] while a double knockout of SULF1 results in neonatal lethality, similar to that seen in EXT1 knockouts [39] although probably through a different mechanism.

When bound to the basal membrane, HSPGs mediate cell-cell and cell-ECM interactions [40–43] with the HS chains acting as receptors or co-receptors triggering cell responses such as proliferation, adhesion, migration and signalling [44, 45]. Two major membrane bound families of HSPG core proteins are the four transmembrane syndecans (SDC; SDC1-4 [46];) and the six glycosyl-phosphatidylinositol (GPI) anchored glypicans (GPC; GPC1-6 [44, 47];). While SDC1 can carry other GAG chains, it predominantly carries HS chains [48] and is a known binding partner of FGF-2 [49]. GPCs carry only HS chains with attachment points close to the cell surface [50]. They are known to have a role in cell proliferation and differentiation and interact with the Wnt signalling pathway. In addition, GPCs have also been suggested to have a role in the inflammatory response [51].

One promising non-immune region, not previously identified in MS genome linkage screens, was 13q31 [52]. One GWAS analysed over 500,000 SNPs in 978 MS cases and 883 control samples and identified the HSPG GPC5 as a novel candidate gene for increased risk in MS [23]. A follow-up study genotyped an additional 974 MS affected individuals with similar results obtained for the GPC5 region [23]. A subsequent study investigating the GPC5 and GPC6 genes in susceptibility to MS determined their strongest association to be a specific GPC5 SNP (rs9523762) [51].

With the genetics of MS not completely understood, genes found to have a modest effect may provide potential new markers of therapy. Previous studies have identified GPCs in active MS plaques [53] and have associated these genes with disease. In contrast, little research has been conducted to elucidate the genetic involvement of SDCs, or HS chain modification enzymes, in MS development and progression. However, there is sufficient evidence to suggest involvement of these genes through their participation in cellular signalling. Indeed, FGF-2, a known signalling partner of the SDCs, has a key role in the regulation of differentiation and proliferation and therefore may have a role in remyelination [49].

To further investigate the role of HSPGs in MS susceptibility, this study set out to replicate previously identified GWAS SNP associations (GPC5, GPC6, 13q31-32 [52]) and to extend our knowledge of the potential involvement of a number of related genes in an Australian Caucasian case-control population. We investigated polymorphisms within HS chain modification enzymes, EXT1 and SULF1, as well as those within HSPG core proteins SDC1, GPC5 and GPC6. Disruption to the critical enzymes responsible for the diverse functions of the HS side chains, or the HSPG core proteins themselves, could have an adverse effect on the progression of MS.

## Methods

### Population DNA

This study utilised a Caucasian (Northern European descent) case-control population. The population consisted of 205 multiple sclerosis (MS) patient samples and 194 age (+/− 5 years) and sex matched control samples. The case samples were then further divided into three disease states: relapsing-remitting (RR), secondary progressive (SP) and primary progressive (PP) MS. The case group consisted of 160 females and 45 males. A summary of the numbers of males and females in each group can be found in Table 1. Genomic DNA was extracted from peripheral blood using a standard salting-out procedure described previously ([54]; Griffith Ethics Approval: 1300000488; QUT Ethics Approval: 1400000502).

**Table 1:** Population demographics

	Total	Age (years)	Males	Age (years)	Females	Age (years)
**Controls**	194	19–96	43	29–70	151	19–96
**Total cases**	205	18–77	45	24–77	160	18–76
**Relapsing remitting (RR)**	100	18–73	15	24–64	85	18–73
**Secondary progressive (SP)**	51	37–73	11	37–70	40	40–73
**Primary progressive (PP)**	54	24–77	19	28–77	35	24–76

DNA was analysed for quantity and quality using the Nanodrop (Thermo Scientific, Australia). Where necessary samples were cleaned using standard ethanol precipitation protocols and reanalysed for quantity and quality. Samples were diluted to a working concentration of 20 ng/μL. Aliquots were stored at −20 °C.

### Primers

Primers for EXT1, SULF1 and SDC1 were designed using NCBI primer blast (NCBI). Primers for GPC5 and GPC6 were designed using PyroMark Assay Design Software v2.0 (Qiagen, Australia) for use in pyrosequencing and subsequent analysis. For GPC6, rs9524260, a sequencing primer was included in the design process. Primer sequences can be found in Table 2. Primers were synthesised by Integrated DNA Technologies (IDT), USA.

**Table 2:** Assay details and SNP information including RFLP fragment sizes where appropriate

SNP number	Gene	Forward primers	Reverse primers	Chr	Chr position	Amplicon length (bp)	Variation	T_a_ (°C)	RFLP fragment sizes (bp)	Accession number	Assay type
rs11546829	*EXT1*	5’ ACAGCCCCTTCCTTACCTGT 3'	5’ GGAAGTAAGGTCAGCCAAACC 3'	8	118847782	397	G/A	51	115, 281	NT_008046.16	RFLP
rs2623047	*SULF1*	5’ GGGATGCACAGAAACCCTAA 3'	5’ TGTGGCAAACAGTGAAGAGC 3'	8	70378496	291	C/T	57	212, 78	NT_008183.19	RFLP
rs1131351	*SDC1*	5’ TGCTGTACCGCATGAAGAAG 3'	5’ GCTGTGGTGGAAAGGTCCTA 3'	2	20402380	354	C/G	62	259, 94	NT_015926.15	RFLP
rs7333912	*GPC5*	5’ GGAAACATAACAAAGTTTGCAATC 3’	5’ TGGGGAGGGATAGGAAGATAAA 3’	13	91874131	120	C/G	49	N/A	NT_009952.14	HRM
rs10492503	*GPC5*	5' CTTCAATACTCTTGCTTGAATCGT 3'	5' CCGTAATTTGTGAGATATACCTTC 3'	13	92885097	115	A/T	58	N/A	NT_009952.14	HRM
rs9523787	*GPC5*	5’ TTCCTAGTTGATTGTTGAAGAGA 3’	5’ TGTAACCTTGATTTTCTTTCTAGT 3’	13	93363760	105	G/T	49	N/A	NT_009952.14	HRM
rs17267815	*GPC6*	5' ATGAGAGGGCTTCCATATAATCAT 3'	5' GGCAACAGTTTTGGAAGAAACA 3'	13	94153058	129	A/G	58	N/A	NT_009952.14	HRM
rs9524260	*GPC6*	5’ GACAGCCAGTGAATGTAGATAGGA 3’	5’ Biotin-CAAATAACAGGAAGCTCAG 3’	13	94513790	105	G/A	56	N/A	NT_009952.14	Pyro
		Sequencing primer	5' CAAATAACAGGAAGCTCAG 3'								

### Polymerase chain reaction

For EXT1 and SULF1, 40 ng of DNA was amplified with 1× PCR buffer, 100 nM each forward and reverse primers (IDT, USA), 200 μM dNTPs (NEB, Australia), 1.75 mM MgCl_2_, 0.5 U GoTaq Flexi DNA Polymerase (Promega, Australia) in a 15-μL reaction. Optimal reaction conditions for SDC1 amplified 40 ng of DNA with 1× PCR buffer, 200 nM each forward and reverse primers, 200 μM dNTPs, 1.75 mM MgCl_2_, 0.5 U GoTaq Flexi DNA polymerase in a 15-μL reaction.

Annealing temperatures (T_a_) for individual assays can be found in Table 2. Cycling conditions for these three SNPs were as follows: an initial denaturation step at 95 °C for 10 min was followed by 35 cycles of 95 °C for 30 s, annealing at T_a_ for 30 s and extension at 72 °C for 30 s. This was followed by a final extension step at 72 °C for 5 min.

PCR products for all SNPs were analysed on a 2% agarose gel in 1× TAE with ethidium bromide at 90 V for at least 30 min to confirm amplification of single PCR products of the correct size. A 100 bp ladder was used alongside the samples for sizing purposes. Fragments were visualised using UV light.

### Restriction fragment length polymorphism

Genotypes for EXT1, SULF1 and SDC1 were determined by RFLP. EXT1 PCR product (7 μL, approximately 1 μg DNA) was digested with 0.5 U Cac8I enzyme, 1× NEB reaction buffer 4 in a total volume of 15 μL. Digestion occurred overnight at 37 °C followed by an inactivation step at 65 °C for 20 min. Digest products were then analysed on a 3% agarose gel in 1× TAE with ethidium bromide at 90 V for 45 min. DNA fragments were visualised under UV light. A 100 bp ladder was used alongside digest products for sizing purposes. The enzyme Cac8I recognises the sequence GCNNGC. The presence of the wild-type G allele allows for digestion producing fragments of 281 bp and 115 bp. The homozygous variant (AA) remained uncut with a single band at 397 bp.

SULF1 and SDC1 PCR products (7 μL, approximately 1 μg DNA) were digested with 1 U PspGI enzyme, 1× NEB reaction buffer 4 in a 15-μL reaction. Samples were digested at 75 °C for 16 h. No inactivation step was required. SULF1 digest products were analysed on a 3% agarose gel as for EXT1. SDC1 digest products were assessed on a 4% agarose gel in 1× TAE with ethidium bromide at 75 V for 1 h along with a 100 bp ladder for sizing purposes. Fragments were visualised under UV light. The enzyme PspGI recognises the sequence CCWGG. The digestion of SULF1 PCR products produces bands at 212 bp and 78 bp in the presence of the wild-type C allele while the homozygous variant remained uncut with a single band visible at 291 bp. When PCR product from SDC1 was digested, bands were produced at 188 bp, 37 bp and 17 bp regardless of the allele present. In the presence of the wild-type G allele, bands are also produced at 53 bp and 43 bp, while the homozygous variant (CC) contains a single extra band at 96 bp.

### High resolution melt

High resolution melt (HRM) analyses were performed on the Rotor Gene™ 6000 (Corbett, Australia), Rotor-Gene® Q (Qiagen, Australia) or with the 7900 HT Fast Real-Time PCR System (Applied Biosystems, Australia). All DNA case-control samples were assayed in duplicate. For each SNP, positive control samples were identified from samples not included in the analysis population. Where possible, positive controls representing each of the three genotypes were identified for each SNP.

HRM reaction conditions amplified 40 ng of DNA with 1× reaction buffer, 1.75 mM MgCl_2_, 100 nm each of forward and reverse primers, 100 nM dNTPs, 50 μM Syto®9 and 0.5 U GoTaq Hot Start DNA polymerase in a 15-μL reaction. Cycling conditions on the ABI 7900 HT system included an initial 2-min hold at 50 °C followed by denaturation and HotStart polymerase activation step of 95 °C for 10 min. This was followed by 40 cycles of 95 °C for 15 s and T_a_ for 1 min. Finally, products were melted between 60 °C and 90 °C to produce the melt curves required to differentiate between genotypes. The Rotor Gene 6000 system utilised the following cycling conditions: denaturation and HotStart polymerase activation at 95 °C for 10 min followed by 40 cycles of 95 °C for 5 s, T_a_ for 10s, and a final melt between 70 °C and 90 °C. The ABI 7900 HT system was used to genotype rs7333912 and rs9523787 (GPC5) while the Rotor Gene 6000 system was used for genotyping rs10492503 (GPC5) and rs17267815 (GPC6).

### Pyrosequencing

Pyrosequencing was performed as per the manufacturer’s instructions on the Pyromark Q24 (Qiagen, Australia). Briefly, 15 μL of PCR product was combined with 2 μL of Streptavidin Sepharose beads (GE Healthcare, Australia), 40 μL of binding buffer (10 mM Tris-HCl, 2 M NaCl, 1 mM EDTA, 0.1% Tween™ 20, pH 7.6) in a reaction volume of 80 μL. Amplicons were denatured (denaturation buffer: 0.2 M NaOH) and washed with washing buffer (10 mM Tris-Acetate) and 70% ethanol. The clean biotin-labelled amplicons were transferred to a sequencing plate containing 0.3 μM sequencing primer diluted in annealing buffer (20 mM Tris-Acetate, 5 mM MgAc_2_) and denatured for 2 min at 80 °C. Samples were loaded into the PyroMark Q24 chamber for analysis. The reagent cartridge was loaded with appropriate volumes of dNTPs and Enzyme and Substrate mix (Qiagen) as determined by proprietary Q24 equipment software.

### Sequencing validation

To verify the results obtained from the HRM genotyping represent the three different genotypes, positive controls and examples of each genotype from the population samples were sequenced. This was performed by Sanger sequencing using the BigDye® Terminator (BDT) v3.1 Cycle sequencing kit (Thermo Fisher Scientific, Australia). Briefly, PCR product was cleaned with ExoSAP-IT® (Affimetrix) following manufacturers protocols. PCR product concentration was estimated and adjusted for optimal sequencing conditions. Following the BDT reaction, the samples were then cleaned using a standard ethanol precipitation method, dried and resuspended in water. Forward and reverse reactions for each sample were prepared. Samples were then loaded into a 96-well plate followed by separation on a four capillary 3130 genetic analyser system (Thermo Fisher Scientific, Australia). The results were later analysed with AB Sequencing Analysis Software v5.3.

### Statistical analysis

Genotype and allele frequencies were calculated using a standard counting method. Populations were analysed by Hardy-Weinberg equilibrium (HWE) and chi-square tests. A significance level of *p* < 0.05 was used. Where allele or genotype analysis was significant, the population was stratified by either disease type or sex and reanalysed. Global *p* values were adjusted for multiple testing using the Benjimini-Hocherg and Bonferroni methods. Corrections were conducted in R v3.3.0 and Rstudio v0.99.896. Corrected *p* values are presented in results tables.

For the GPC5 and GPC6 SNPs within the chromosome 13 risk region, linkage disequilibrium (LD) analysis was preformed using Haploview v4.2.

## Results

We examined eight single nucleotide polymorphisms (SNPs) within HSPG initiation and modification enzyme (*EXT1* and *SULF1)* and HSGP core protein (*SDC1*, *GPC5* and *GPC6)* genes in an Australian case-control population for their role in MS susceptibility. Genotype and allele frequencies were also compared to results from the HapMap CEU reference population. The chromosomal region of 13q31-32, where GPC5 and GPC6 are located, has been previously identified in a GWAS as showing a significant association with genetic susceptibility of multiple sclerosis (MS). We aimed to investigate the role of these genes as well as the potential role of enzymes responsible for the modification of the HS chains attached to these and other core proteins. This study investigated eight SNPs in five genes. Differences in final population numbers successfully genotyped for each SNP analysis are due to variation in assay efficiency as well as DNA availability.

### Modification enzymes

EXT1 and SULF1 enzymes initiate and modify HS side chains attached to core proteins. The action of these enzymes determines the final length and sulfation pattern of the side chain and mediate signalling interactions (eg. growth factors). They are critical for HSPG synthesis and any mutation could result in an autosomal dominant disorder [33].

#### EXT1, rs11546829

No significant association was found between the rs11546829 SNP in EXT1 and MS. Both case and control populations followed HWE and allele and genotype frequencies matched the HapMap CEU reference population. When the population was stratified by disease type and further analysed, there was no association found with disease state. Results are summarised in Table 3.

**Table 3:** Genotype and allele frequencies of EXT-1 SNP (rs11546829) within the case-control MS population which is further subdivided into disease states. Corrected *P* values using the Benjimini-Hochberg (P_BH_) and Bonferroni (P_Bon_) methods are presented below the uncorrected *P* value

	EXT1-829	rs11546829							
Polymorphism	Genotypes					Alleles			
Group	GG (%)	GA (%)	AA (%)	*P*(P_**BH**_, P_**Bon**_)	HWE	G (%)	A (%)	***P***	OR (95% CI)
**MS total cases (*n*= 176)**	80 (45.5)	78 (44.3)	18 (10.2)	0.45 (0.72, 1)	0.87	238 (67.6)	114 (32.4)	0.75	0.95
**PP case (*n*= 49)**	22 (44.9)	22 (44.9)	5 (10.2)	0.67		66 (67.3)	32 (32.7)	0.87	0.96
**SP case (*n*= 43)**	21 (48.8)	19 (44.2)	3 (7)	0.45		61 (70.9)	25 (29.1)	0.44	0.81
**RR case (*n*= 84)**	37 (44)	37 (44)	10 (12)	0.72		111 (66.1)	57 (51.4)	0.94	1.02
**Control (*n*= 134)**	63 (47)	52 (38.9)	19 (14.2)	–	0.13	178 (66.4)	90 (33.6)	–	–
**HapMap CEU (%)**	58.3	36.7	5			76.7	23.3		

#### SULF1, rs2623047

No significant association was found between rs2623047 in SULF1 and MS. HWE was observed in both case and control populations. Allele and genotype frequencies matched the HapMap CEU reference population. Further analysis of the stratified populations revealed no significant association with disease type. Results are summarised in Table 4.

**Table 4:** Genotype and allele frequencies in the case-control MS population for the SULF-1 SNP (rs262347) further subdivided into disease states. Corrected *p* values using the Benjimini-Hochberg (P_BH_) and Bonferroni (P_Bon_) methods are presented below the uncorrected *P* value

	SULF1	rs2623047							
Polymorphism	Genotypes					Alleles			
Group	CC (%)	CT (%)	TT (%)	***P*** (P_**BH**_, P_**Bon**_)	HWE	C (%)	T (%)	***P***	OR (95% CI)
**MS total cases (*n*= 190)**	21 (11.1)	84 (44.2)	85 (44.7)	0.94 (0.94, 1)	0.97	126 (33.2)	254 (66.8)	0.94	1.01
**PP case (*n*= 50)**	7 (14)	20 (40)	23 (46)	0.68		34 (34)	66 (66)	0.92	0.97
**SP case*(n*= 46)**	8 (17.4)	18 (39.1)	20 (43.5)	0.40		34 (37)	58 (63)	0.53	0.86
**RR case (*n*= 94)**	6 (6.4)	46 (48.9)	42 (44.7)	0.53		58 (30.9)	130 (69.1)	0.54	1.13
**Control (*n*= 172)**	18 (10.5)	79 (45.9)	75 (43.6)	–	0.68	115 (33.4)	229 (66.6)	–	–
**HapMap CEU (%)**	15.9	51.3	32.7			41.6	58.4		

### Core proteins

#### Syndecan-1

SDC1 is known to have a role in various cancers, including breast cancer [56] through its role in cell adhesion, migration and proliferation. Through its interaction with FGF-2, SDC1 has also been proposed to have a role in remyelination [49]. Evidence suggests that SDC1 may be associated with MS through enhanced expression of TGF-β in MS lesions that may lead to increased expression of SDC1 [55]. A link between SDCs and the innate immune response has also been postulated as these HSPGs have been observed in injured tissues, regulating the accompanying inflammatory response [48], suggesting a link to the inflammatory response seen in MS patients.

#### SDC1, rs1131351

A positive association between the rs1131351 SNP in SDC1 and MS was identified. Both case and control populations followed HWE. When compared to the HapMap CEU, reference population allele and genotype frequencies were similar. Allele and genotype frequencies showed significant differences between the case and control populations. When the population was examined by disease state, significant association for the SDC1 SNP was observed at the allelic level for RRMS and PPMS for the whole population. There was no significant association with SPMS. Results are summarised in Table 5.

**Table 5:** Results by disease state for SDC1 SNP (rs1131351) and of the MS case-control population. Corrected *P* values using the Benjimini-Hochberg (P_BH_) and Bonferroni (P_Bon_) methods are presented below the uncorrected *P* value

	SDC 1	rs113151							
Polymorphism	Genotypes					Alleles			
Group	GG (%)	GC (%)	CC (%)	***P*** (P_**BH**_, P_**Bon**_)	HWE	G (%)	C (%)	***P***	OR (95% CI)
**MS total cases (*n*= 160)**	31 (19.4)	83 (51.9)	46 (28.8)	**0.02** (0.08, 0.16)	0.55	145 (45.3)	175 (54.7)	**0.004**	1.59
**PP case (*n*= 45)**	8 (17.8)	24 (53.3)	13 (28.9)	0.11		40 (44.4)	50 (55.6)	**0.04**	1.65
**SP case (*n*= 39)**	8 (20.5)	19 (48.7)	12 (30.8)	0.15		35 (44.9)	43 (55.1)	0.06	1.62
**RR case (*n*= 76)**	15 (19.7)	40 (52.6)	21 (27.6)	0.09		70 (46.1)	82 (53.9)	**0.03**	1.55
**Male (*n*= 31)**	7 (22.6)	17 (54.8)	7 (22.6)	0.58		31 (50)	31 (50)	0.32	1.32
**Female (*n*= 129)**	24 (18.6)	66 (51.2)	39 (30.2)	**0.01**		114 (44.2)	144 (58.8)	**0.003**	1.67
**Total control (*n*= 145)**	46 (31.7)	73 (50.3)	26 (17.9)	–	0.75	165 (56.9)	125 (43.1)	–	
**HapMap CEU (%)**	50	31	19			65.5	34.5		

The population was further analysed by sex, where SDC1 showed a further significant association in the female MS population (Table 5). The female population was further stratified by disease state. A significant association was observed in females with PPMS at both the genotype and allelic level and female RRMS cases at the allelic level only with no observed significance with genotypes. The female SPMS group demonstrated no significant association at either allele or genotype level and MS. Results are summarised in Table 6.

**Table 6:** Female results by disease state for SDC1 SNP (rs1131351)

	SDC1	rs1131351						
Polymorphism	Genotypes				Alleles			
Group	GG (%)	GC (%)	CC (%)	***P***	G (%)	C (%)	***P***	OR (95% CI)
**Female MS case**								
**PP (*n*= 31)**	3 (9.68)	17 (54.8)	11 (35.5)	**0.02**	23 (37.1)	39 (62.9)	**0.005**	2.24
**SP (*n*= 28)**	7 (25)	13 (46.4)	8 (28.6)	0.41	27 (48.2)	29 (51.8)	0.23	1.42
**RR (*n*= 70)**	14 (20)	36 (51.4)	20 (28.6)	0.09	64 (45.7)	76 (54.3)	**0.03**	1.57
**Total control (*n*= 145)**	46 (31.7)	73 (50.3)	26 (17.9)	–	165 (56.9)	125 (43.1)	–	–
**HapMap CEU (%)**	50	31	19		65.5	34.5		

#### Glypican-5

The three GPC5 SNPs (rs7333912, rs10492503 and rs9523787) investigated in this study have previously been significantly associated with MS in Caucasian European populations [51, 57]. Another GPC5 SNP (rs9523762), not reported here, was found to be positive in one study [23] while another study identified moderate LD between it and rs9523787 but did not find it to be individually significant [51]. Indeed, in this current study, we also investigated this SNP but have not presented the results as analysis revealed significant deviation from HWE. As this Australian population consists of Caucasian ancestors, we performed an associative study with these SNPs in an Australian Caucasian population to see if the association could be replicated in this cohort. Differences between our results and previous studies may be due to the more mixed heritage of the Australian Caucasian population compared with the purer northern European Caucasian populations previously examined.

#### GPC5, rs7333912

The GPC5 SNP, rs7333912, is an intergenic SNP, located at 13q31-32 approximately 150,000 bp upstream of the GPC5 gene on chromosome 13. This variation is a C/G polymorphism with no homozygous variants (GG) observed either in our population or in the HapMap CEU reference population. The case and control populations were found to be in HWE with no significant association identified between this SNP and MS. When the population was stratified and analysed by disease state, no significant association was found with disease type. Results are summarised in Table 7.

**Table 7:** Results for GPC5, rs7333912. Corrected *P* values using the Benjimini-Hochberg (P_BH_) and Bonferroni (P_Bon_) methods are presented below the uncorrected *P* value

	GPC5	rs7333912							
Polymorphism	Genotypes					Alleles			
Group	CC (%)	GC (%)	GG (%)	***P*** (P_**BH**_, P_**Bon**_)	HWE	C (%)	G (%)	***P***	OR (95% CI)
**MS total cases (*n*= 205)**	195 (95.1)	10 (4.9)		0.768 (0.878, 1)	0.72	400 (97.6)	10 (2.4)	0.771	1.15
**PP case (*n*= 54)**	52 (96.3)	2 (3.7)				106 (98.1)	2 (1.9)	0.859	0.87
**SP case (*n*= 51)**	47 (92.2)	4 (7.8)				98 (95.1)	4 (3.9)	0.189	2.11
**RR case (*n*= 100)**	96 (96.0)	4 (4.0)				196 (98.0)	4 (2.0)	0.667	0.75
**Control (*n*= 188)**	180 (95.7)	8 (4.3)			0.766	368 (97.9)	8 (2.1)		
**HapMap CEU (%)**	99.1	0.9				99.6	0.4		

#### GPC5, rs10492503

GPC5-rs10492503 is located in the same intron as rs9523787 (intron7-8) within the 13q31-32 MS susceptibility locus that has previously been associated with MS [52]. In our study, both case and control populations observed HWE. A significant association was identified between this SNP and disease at both the genotype and allele level. When further analysed by disease state, further significant associations were determined in the SPMS group at the allelic level and RRMS case group at the both the genotypic and allelic level. Stratification by sex determined a significant association between this SNP and the female case group at both genotype and allele level. Results are summarised in Table 8. The female case group was further analysed by disease state. A significant association was found at both genotypic and allelic level between the SNP and female SPMS and RRMS disease subtypes. These results are summarised in Table 9.

**Table 8:** Results for GPC5, rs10492503. Corrected *P* values using the Benjimini-Hochberg (P_BH_) and Bonferroni (P_Bon_) methods are presented below the uncorrected *P* value

	GPC5	rs10492503							
Polymorphism	Genotypes					Alleles			
Group	AA (%)	AT (%)	TT (%)	*p* (P_**BH**_, P_**Bon**_)	HWE	A (%)	T (%)	*P*	OR (95% CI)
**MS total cases (*n*= 204)**	67 (32.8)	98 (48.1)	39 (19.1)	**0.016** (0.08, 0.128)	0.767	232 (56.8)	176 (43.2)	**0.0079**	1.50
**PP case (*n*= 53)**	24 (45.3)	24 (45.3)	5 (9.4)	0.492		72 (67.9)	34 (32.1)	0.781	0.94
**SP case (*n*= 51)**	13 (25.5)	31 (60.8)	7 (13.7)	**0.0098**		57 (55.9)	45 (44.1)	0.0519	1.56
**RR case (*n*= 100)**	30 (30)	43 (43)	27 (27)	**0.0067**		103 (52.5)	97 (48.5)	**0.0006**	1.87
**Male (*n*= 44)**	14 (31.8)	21 (47.7)	9 (20.5)	0.560		49 (55.7)	39 (44.3)	0.299	1.42
**Female (*n*= 160)**	53 (33.1)	77 (48.1)	30 (18.8)	**0.0286**		183 (57.2)	137 (42.8)	**0.0148**	1.52
**Control (*n*= 164)**	78 (47.6)	62 (37.8)	24 (14.6)		0.052	218 (66.5)	110 (33.5)		
**HapMap CEU (%)**	38.3	51.7	10			64.2	35.8

**Table 9:** Female results by disease state for GPC5, rs10492503. Significance for the PP case subgroup is suggestive only as cell counts fell below the minimum required for chi-squared testing (*n* < 5)

	GPC5	rs10492503						
Polymorphism	Genotypes				Alleles			
Group	AA (%)	AT (%)	TT (%)	***p***	A (%)	T (%)	***P***	OR (95% CI)
**Female MS case**								
**PP (*n*= 35)**	17 (48.6)	16 (45.7)	2 (5.7)	0.3358	50 (71.4)	20 (28.6)	0.4847	0.81
**SP (*n*= 40)**	9 (22.5)	25 (62.5)	6 (15.0)	**0.0087**	43 (53.8)	37 (46.2)	**0.030**	1.75
**RR (*n*= 85)**	27 (31.8)	36 (42.4)	22 (25.9)	**0.0245**	90 (52.9)	80 (47.1)	**0.0032**	1.81
**Total control (*n*= 132)**	64 (48.5)	49 (37.1)	19 (14.4)	–	177 (67.1)	87 (32.9)	–	–
**HapMap CEU (%)**	38.3	51.7	10		64.2	35.8		

#### GPC5, rs9523787

This variation is located toward the 3’ end of the gene in intron 7-8, the same intron that contains rs10492503 [52]. GPC5-rs9523787 had previously been associated with MS and to be in modest LD with a variation located close by, rs9523762 [51]. We were unable to replicate either of these findings. Both case and control populations followed HWE and we found no significant association between this variation and the MS population. Populations stratified by disease type also showed no significant association. Results are summarised in Table 10.

**Table 10:** Results for GPC5, rs9523787. Corrected *P* values using the Benjimini-Hochberg (P_BH_) and Bonferroni (P_Bon_) methods are presented below the uncorrected *P* value. Significance measures are suggestive only as cell counts fell below the minimum required to perform chi-square analysis in the disease subgroups (*n* < 5)

	GPC5	rs9523787							
Polymorphism	Genotypes					Alleles			
Group	GG (%)	GT (%)	TT (%)	***P*** (P_**BH**_, P_**Bon**_)	HWE	G (%)	T (%)	***P***	OR (95% CI)
**MS total cases (*n*= 205)**	146 (71.2)	54 (26.3)	5 (2.5)	0.609 (0.812, 1)	0.998	346 (84.4)	64 (15.6)	0.464	0.87
**PP case (*n*= 54)**	37 (68.5)	17 (31.5)	0 (0)	0.558		91 (84.3)	17 (15.7)	0.660	0.88
**SP case (*n*= 57)**	40 (78.4)	9 (17.7)	2 (3.9)	0.153		89 (87.3)	13 (12.7)	0.246	0.69
**RR case (*n*= 94)**	69 (69.0)	28 (28.0)	3 (3.0)	0.811		166 (83.0)	34 (17.0)	0.868	0.96
**Control (*n*= 188)**	126 (67.0)	58 (30.9)	4 (2.1)		0.366	310 (82.4)	66 (17.6)		
**HapMap CEU (%)**	64.6	27.4	8			78.3	21.7		

#### Glypican-6

GPC6 has previously been implicated in MS [51]. In that study, 22 SNPs were analysed with only three showing a significant association with disease (GPC5-rs7333912, GPC6-rs17267815, GPC6-rs12876985). Only GPC5-rs7333912 and GPC6-rs17267815 were included in this study. The second GPC6 SNP investigated in this study was previously reported to be associated with primary sclerosing cholanigitis (PSC) [58]. GPC6 is located in the chromosome region of 13q31 neighbouring the risk region identified in genome wide screens [52].

#### GPC6, rs17267815

Of 22 SNPs analysed by Lorentzen and colleagues, this SNP (GPC6-rs17267815) showed the greatest significance [51] with MS. In our study, no significant association was identified in the total case group versus controls. However, when the cases were analysed by disease state, we found a significant association between the RRMS case group and the SNP at both the genotype and allelic level. Results are summarised in Table 11. There was no significance when stratified by sex. However, when the population was stratified by both disease and sex, an association was found at both the genotype and allelic level for the male RRMS subgroup. This is, however, only suggestive as the sample numbers for the homozygous variant genotype fell below the minimum required for reliable chi-square testing (*n* < 5). These results are summarised in Additional file 1: Table S1.

**Table 11:** Results for GPC6, rs17267815. Corrected *P* values using the Benjimini-Hochberg (P_BH_) and Bonferroni (P_Bon_) methods are presented below the uncorrected *P* value

	GPC6	rs17267815							
Polymorphism	Genotypes					Alleles			
Group	AA (%)	AG (%)	GG (%)	***P*** (P_**BH**_, P_**Bon**_)	HWE	A (%)	G (%)	***P***	OR (95% CI)
**MS total cases (*n*= 205)**	46 (22.4)	105 (51.2)	54 (26.4)	0.0797 (0.213, 0.638)	0.71	197 (48.1)	213 (51.9)	0.118	0.79
**PP case (*n*= 54)**	10 (18.5)	26 (48.2)	18 (33.3)	0.691		46 (42.6)	62 (57.4)	0.925	0.98
**SP case (*n*= 51)**	8 (15.7)	29 (56.9)	14 (27.4)	0.161		45 (51.7)	42 (48.3)	0.719	0.92
**RR case (*n*= 100)**	28 (28.0)	50 (50.0)	22 (22.0)	**0.039**		106 (51.7)	94 (48.3)	**0.017**	0.64
**Male (*n*= 45)**	14 (31.1)	20 (44.4)	11 (24.4)	0.1436		48 (53.3)	42 (46.7)	**0.0386**	0.51
**Female (*n*= 160)**	32 (20.0)	85 (53.1)	43 (26.9)	0.1154		149 (46.6)	171 (53.4)	0.5095	0.89
**Control (*n*= 145)**	31 (21.4)	60 (41.4)	54 (37.2)		0.069	205 (54.5)	171 (45.5)		
**HapMap CEU (%)**	26.5	54	19.5			53.5	46.5		

#### GPC6, rs9524260

Located within the 13q31 risk region, rs9524260 is an intronic SNP. Both case and control populations were found to be in HWE with no significant association found between this variation and MS. There was also no significant association when the population was stratified by disease type or sex. The results are summarised in Table 12.

**Table 12:** Results for GPC6, rs9524260. Corrected *P* values using the Benjimini-Hochberg (P_BH_) and Bonferroni (P_Bon_) methods are presented below the uncorrected *P* value

	GPC6	rs9524260							
Polymorphism	Genotypes					Alleles			
Group	GG (%)	GA (%)	AA (%)	***P*** (P_**BH**_, P_**Bon**_)	HWE	G (%)	A (%)	***P***	OR (95% CI)
**MS total cases (*n*= 197)**	76 (38.6)	90 (45.7)	31 (15.7)	0.236 (0.472, 1)	0.613	242 (61.4)	152 (38.6)	0.974	1.00
**PP case (*n*= 53)**	22 (41.5)	21 (39.6)	10 (18.9)	0.146		65 (61.3)	41 (38.7)	0.967	1.01
**SP case (*n*= 50)**	16 (32.0)	24 (48.0)	10 (20.0)	0.296		56 (56.0)	44 (44.0)	0.316	1.26
**RR case (*n*= 94)**	38 (40.4)	45 (47.9)	11 (11.7)	0.607		121 (64.4)	67 (35.6)	0.516	0.89
**Control (*n*= 182)**	63 (34.6)	98 (53.8)	21 (11.6)		0.064	224 (61.5)	140 (38.5)		
**HapMap CEU (%)**	36.3	54.9	8.8			63.7	36.3		

### LD analysis of GPC5 and GPC6 SNPs

**Fig. 1:**
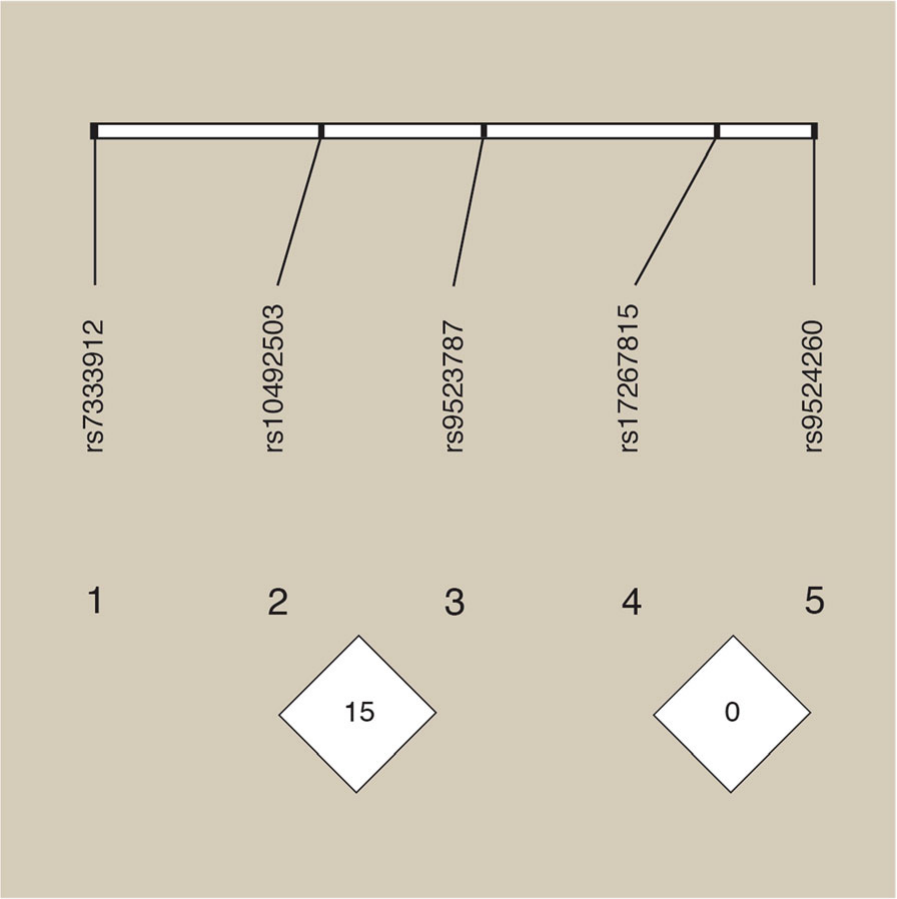
LD Plot from GPC5/GPC6 haplotype analysis. The figure shows there is no LD between these SNPs in an Australian Caucasian MS population. This analysis was unable to replicate the positive associations and LD found in previous studies

GPC5 and GPC6 markers were analysed for LD using Haploview v4.2. No LD was observed in this population. The highest D’ value determined was between rs10492503 and rs9523787 in GPC5 with D’ = 0.15. The LD plot can be seen in Fig. 1.

## Discussion

Multiple sclerosis (MS) is a chronic inflammatory disease of the central nervous system (CNS) with a presumed autoimmune origin, triggered by genetic and environmental risk factors. The aetiology of MS is unknown, and the pathology is not well understood. In addition to those SNPs identified as significant in a 2009 GWAS [23], we investigated two SNPs in enzymes responsible for the initiation and modification of the side chain characteristic of HSPGs (EXT1, SULF1) and another HSPG core protein, SDC1. No significant difference was observed in our MS population in relation to the EXT1 and SULF1 SNPs examined. Further analysis revealed no association with disease subtype. However, in this study, we did identify significant associations with SDC1, GPC5 and GPC6 polymorphisms. Overall, analysis by disease subtype maintained this significance, as did analysis by sex.

While this study may suggest that no association exists between EXT1 and MS, previous studies have indicated a strong expression of EXT1 in the developing brain [32]. Additionally, it has been suggested that EXT1 correlates with the sites of active neuron generation [32]. Prenatally EXT1 has been localised in the neuroepithelial cells, which surround the lateral ventricles, cerebral cortex and hippocampus. However, in the postnatal stage, EXT1 is expressed in the cerebellum, which may correlate with the symptoms seen in MS such as ataxia [32]. In a murine model, complete abrogation of EXT1 results in embryonic lethality during gastrulation due to the absence of HS [34]. EXT1 alone is able to polymerise GAG chains for attachment to PG core proteins [59, 60]; however, both EXT1 and EXT2 are required for in vivo HS chain elongation [61].

SULF1 has been suggested to have a role in the brain; however, deficiencies in SULF1 have been associated with developmental abnormalities such as decreased body mass and subtle kidney and bone defects [34]. SULF1 has also been linked to tumour suppressor functions as it has been reported to be downregulated in some cancers [37].

The process of HS chain biosynthesis requires the action of enzymes such as SULF1 to generate complex sulfation patterns through the addition and removal of sulfation sites. Successful binding of growth factors to GAG chains for signalling pathway activation requires specific sulfation patterns on these side chains. The interaction between HS and FGF-2 is a well-documented example where HS requires 2-*O* sulfation to be able to bind to FGF-2. Without this binding, cellular proliferation and differentiation are inhibited. Although these two SNPs in EXT1 and SULF1 showed no significance in our population of moderate size, further investigation should be undertaken with a larger cohort before excluding the possibility of their involvement in MS susceptibility.

In the first of the HSPG core proteins examined (SDC1), we found a significant association between the SNP, rs1131351 and MS. This association revealed a stronger link between the SNP and females suffering from early-onset forms of the disease (PPMS, RRMS). SPMS occurs 8–20 years after RRMS onset [14] with the negative association seen here with this disease state suggestive of a role for SDC1 in the initiation of disease. Females with PPMS and the minor allele of SDC1 have more than double the risk (OR = 2.24) of developing MS than controls. In patients suffering from RRMS, this increase in risk is approximately 1.5 times (OR = 1.57). This could be due to the fact that PPMS seems to be more aggressive during onset when compared with RRMS. Even though they are both classified as onset stages of the disease, RRMS can progress to SPMS, with reversible damage occurring in this stage, while PPMS damage is irreversible and the symptoms are generally more detrimental (reviewed in [62, 63].

Active MS lesions are characterised by an influx of inflammatory cells and a decrease of chondroitin sulfate proteoglycans (CSPGs) [55]. Furthermore, white matter-associated PGs have been known to accumulate in macrophages, suggesting that CSPGs are phagocytosed with myelin or their breakdown products [55]. SDC1 contains ser-gly sequences that may serve as an attachment site for chondroitin sulfate (CS) [48] while also carrying HS chains. A mutation in SDC1 may contribute to activation of the macrophages causing phagocytosis, subsequently leading to a reduction in SDC1 in MS patients. In addition, TGF-β along with FGF-2 have been linked to enhanced expression of SDC1 [48]. Enhanced expression of TGF-β has been observed in MS lesions causing matrix deposition by the promotion of transcription genes and suppression of degrading enzymes [55]. FGF-2 has been associated with the survival, proliferation and migration of oligodendrocyte precursors leading to the promotion of remyelination [64]. This contradicts the mechanism of neurodegeneration seen in MS patients; however, FGF-2 could be a survival mechanism established to reverse the damage particularly in relapsing and remitting MS patients, through its binding partners other than SDC1.

In addition to the SDC1-FGF-2/TGF-β signalling mechanisms, TNF-α has been demonstrated to decrease SDC1 expression in cultured endothelial cells [48]. TNF-α has been shown to be involved in the inflammatory response [65] and could be involved in the process mimicking the early stages of MS where the breakdown of the blood-brain barrier allows inflammatory cells to cross into the brain and contribute to demyelination and axonal damage [55].

In this study, we aimed to replicate and build on results from a number of previous GWAS and replication studies using an Australian case-control population. These earlier results implicated GPC5 and GPC6 SNPs in MS. Our analysis of three GPC5 and two GPC6 SNPs also identified significant associations between these genes and MS susceptibility. Comparisons between these previous studies and our current study are summarised in Table 13. GPC5-rs10492503 showed a significant association in the total disease population. When analysed further, we found significant associations with two disease states (SPMS and RRMS) and in the total female population and the female SPMS and RRMS subtypes. GPC6-rs17267815 showed a minor significant association within the RRMS subtype only. Further analysis suggested that this association was due to the male RRMS subgroup; however, due to low sample numbers, once the population was stratified, significance values are suggestive only.

**Table 13:** Comparison of significance obtained in this study compared to previous GWAS. *P* values from GWAS presented as from the original paper. P_un_ = uncorrected *P* value; P_C_ = corrected *p* value. Baranzini et al. presented their significance as adjusted log *P* values

		Genotype (%)			Allele (%)		Current study			GWAS significance		
Gene	SNP	Hom (%)	Het (%)	Var (%)	Allele 1	Allele 2	*P* value	Corrected B-H	Corrected Bonferroni	Cenit 2009 P_un_ (P_C_)	Baranzini 2009 Adj Log *p* value	Lorentzen 2010 P_un_
**GPC5**	rs7333912	95.1	4.9	0	97.6	2.4	0.76	0.878	1			0.02
**GPC5**	rs10492503	32.8	48.1	19.1	56.8	43.2	**0.016**	0.08	0.128	0.016 (0.096)		
**GPC5**	rs9523787	71.2	26.3	2.5	84.4	15.6	0.069	0.812	1			0.0002
**GPC5**	rs9523762	Did not follow Hardy-Weinburg equilibrium									5.155	
**GPC6**	rs17267815	22.4	51.2	26.4	48.1	51.9	0.0797	0.213	0.638			0.03
**GPC6**	rs9524260	38.6	45.7	15.7	61.4	38.6	0.236	0.472	1			0.10

We identified no LD between the SNPs studied within the previously identified 13q31-32 risk region containing both these genes, nor could we replicate the moderate LD identified previously in GPC5 [51]. All five GPC5 and GPC6 SNPs investigated in this study had previously been identified as significant in large-scale case/control GWAS and replication studies in Norwegian and Spanish populations [23, 51, 52, 57] with varying and often contradictory levels of significance. SNPs reaching significance in one study were not found to be significant in another [23, 51, 53]. Analysis by the disease state of some of these populations determined significant associations with the RRMS subtype [66]. Indeed, in our population, when a significant association was observed in these genes, it was often significant in the RRMS sub-population. This may be due to the mixture of the populations as patients from pure Northern European ancestry have a higher risk of developing MS [8]. While our Australian population is of Caucasian descent, it is not necessarily of purely Northern European origin, potentially explaining some differences between results and levels of significance identified in these studies. In addition, while our results are not strongly significant on their own, they replicate previous studies and support and strengthen the evidence for the involvement of GPC5 and GPC6 in the development and progression of MS.

Many HSPGs and their associated enzymes have been associated with the disease, with both SDC1 and SDC4 showing strong involvement with breast cancer [46, 67, 68]. As yet, the physiological functions of the GPCs, in both normal and pathological conditions, remain poorly understood. However, data here and in other studies suggest an important function for these proteins in cell growth and regulation of division. Celie and colleagues suggest that HSPGs are involved in the inflammatory response and have a regulatory role in leukocyte extravasation [69], a condition synonymous with MS. Other GPCs have been shown to play roles in diseases such as hepatocellular carcinoma (GPC3 [70];) and Simpson-Golabi-Behmel syndrome (GPC3/GPC4 [71];). While the function of GPC5 remains poorly understood, particularly in MS, different GPC polymorphisms have been reported to increase the risk of lung cancer in non-smokers [72] while decreasing the risk of cancer in MS patients, with this reduced cancer risk stemming from the specific GPC gene [73]. In addition, the gene region 13q31-32 containing both GPC5 and GPC6 has also previously been associated with the increased risk of primary sclerosing cholangitis (PSC), a chronic liver disease where a strong association has been identified between the SNP GPC6-rs9524260 and disease [58].

Due to the interaction of the GPCs with several growth factors, chemokines and ECM proteins, there may also be an effect on neural growth and repair [74]. The results of a study by Cenit et al. [57] not only supported a significant association of GPC5*-*rs10492503 with MS but also indicated approximately twice the risk of developing the disease in an individual who has one or more copies of the variant allele [57]. GPC5 has been reported to play an important role during the process of cell division and growth regulation. It is predominantly expressed in foetal tissues, including the brain, lung, liver and kidney. However, it has an exclusive expression in adult tissue in the CNS and in its neurons [71, 75]. This suggests a possible and plausible role for this gene in controlling various neurotropic factors and maintenance of neural function. In our study, we found a significant association of this GPC5 variation with the early-onset form of the disease (RRMS) and also the severe form (SPMS), a progression of the disease characterised by irreversible damage suggesting a role for GPC5 in the progression of MS. GPC5 plays an important role in brain patterning, synapse formation, axon regeneration and guidance. Its expression in the developing brain and the adult CNS (the origin of MS) also support a role for this gene in different disease states.

In PPMS, most of the myelin degradation occurs in the cerebrum and cerebellar cortex of the CNS [1]. Dysfunction of GPC5 could affect cell proliferation and tissue growth. With the cells no longer able to interact with the various positively charged growth factors, this would affect brain patterning, synapse formation and an interruption in axon regeneration. This suggests that abnormal GPC5 may play a role in triggering MS and the subsequent disability experienced by sufferers.

Further evidence supporting this hypothesis is data demonstrating that HSPGs have been identified in the active lesions of MS, where they are thought to be involved in the sequestering of pro-inflammatory chemokines [55]. GPC5 expression and interaction with various growth factors and chemokines likely affect growth and repair of neurons, also influencing the guidance of axons and synapse formation [28, 55, 76]. Indeed, another member of the GPC family—GPC1, has been shown to be required for Schwann cell myelination [77]. With documented involvement of other GPCs, it is plausible allelic variants of GPC5 may affect neuronal repair, axon guidance and new synaptic formation.

The embryonic expression of GPC6 is detected in the ovary, liver and kidneys, while in the adult, it is detected only in the ovary and intestine [78]. More recent emerging evidence for GPC6 may indicate a role for the gene in neural diseases with origins in the CNS, the location of the MS-associated lesions. Overall, the functional role for GPC6 is poorly understood, but this study provides some evidence of a potential role for GPC6 in MS.

## Conclusion

**Fig. 2:**
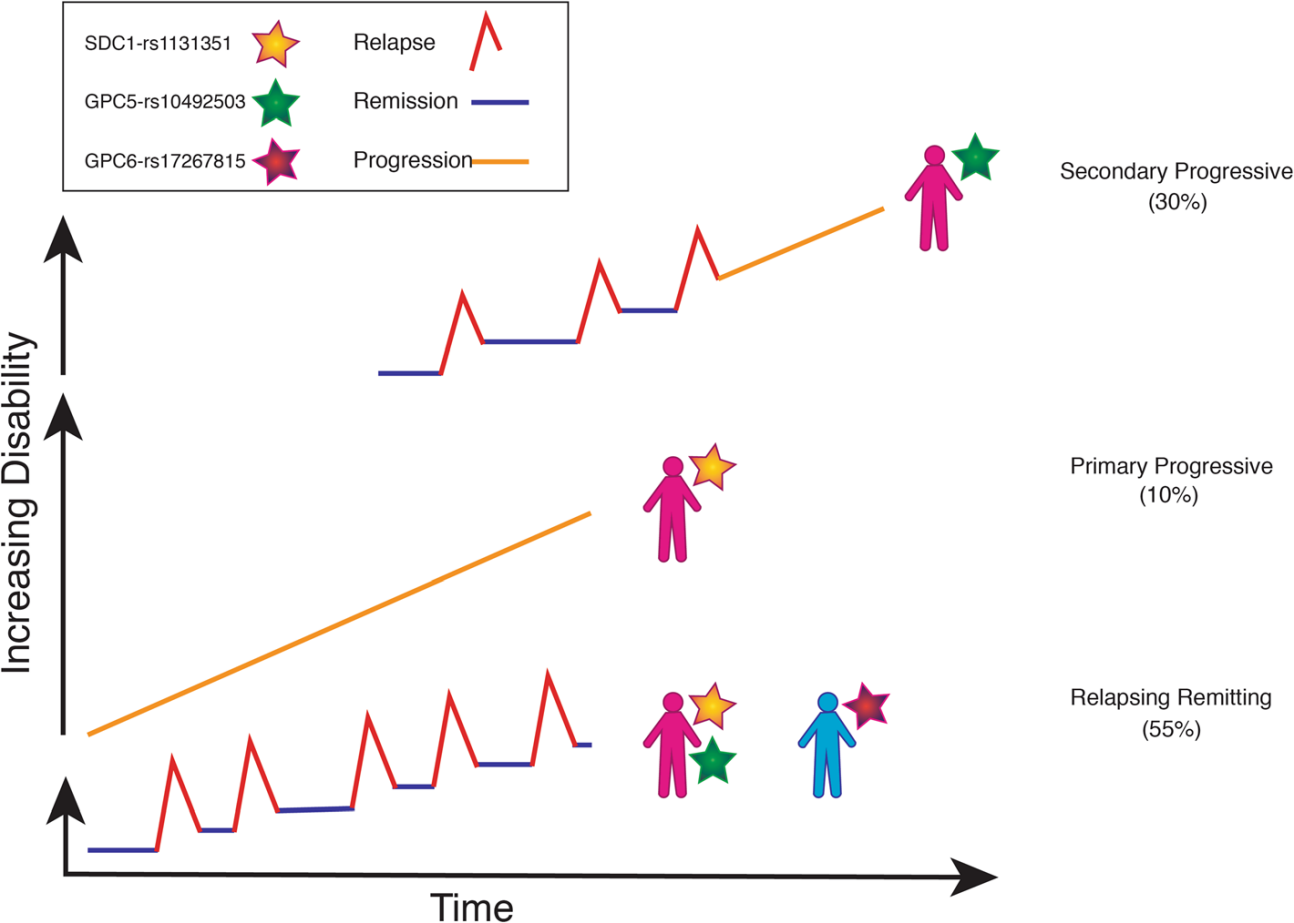
Schematic of disease progression highlighting the involvement of HSPG core protein SNPs. Significant associations between specific HSPG core protein SNPs and disease subtypes are represented by coloured stars as can be seen in the legend. SDC1: yellow; GPC5: green; GPC6: purple. These SNPs are significantly associated with population stratification by gender as represented by coloured people outlines. Female: pink; Male: blue

From this study, we have determined a significant association with the rs1131351 SNP in SDC1, specifically in females suffering from either primary progressive or relapsing-remitting forms of MS. Involvement of SDC1 in the initiation of MS has been suggested through its involvement in the inflammatory response and growth factor interactions. Levels of specific growth factors may vary during MS onset which could be due to dysfunction of HSPGs brought about by their inability to appropriately traffic/sequester growth factors. The specific mechanism of GPC5 and GPC6 involvement in MS has yet to be elucidated. However, a number of genetic studies, this one included, have provided evidence suggesting a role for these genes in the progression of the disease. Evidence already exists for these genes in other diseases utilising similar mechanisms of action. Significant results obtained in this study have been summarised in Fig. 2. This schematic highlights the involvement of specific SNPs in specific disease states as well as whether the SNP is associated with either the male or female subpopulation. Results from this study are by no means conclusive, but they add to the growing body of evidence indicating the involvement of these PGs in the initiation and progression of neurodegenerative diseases. Specifically, this study supports and strengthens evidence suggesting a role for HSPG core proteins, both syndecans and glypicans, in the development and progression of MS.

## Supplementary information

**Additional file 1: Table S1.** Male results by disease state for GPC6, rs17267815.

## Data Availability

All data relevant for this study are included within this manuscript, any further information may be made available on request.

## References

[1] Kutzelnigg A, Lucchinetti CF, Stadelmann C, Bruck W, Rauschka H, Bergmann M, Schmidbauer M, Parisi JE, Lassmann H (2005). Cortical demyelination and diffuse white matter injury in multiple sclerosis. Brain.

[2] Noseworthy JH, Lucchinetti C, Rodriguez M, Weinshenker BG. Multiple sclerosis. N Engl J Med. 2000;343:938–52. 10.1056/NEJM200009283431307 11006371

[3] Liguori M, Marrosu MG, Pugliatti M, Giuliani F, De Robertis F, Cocco E, Zimatore GB, Livrea P, Trojano M. Age at onset in multiple sclerosis. Neurol Sci. 2000;21:825–9. 10.1007/s100720070020 11205357

[4] Tajouri L, Fernandez F, Tajouri S, Detriche G, Szvetko A, Colson N, Csurhes P, Pender MP, Griffiths LR (2007). Allelic variation investigation of the estrogen receptor within an Australian multiple sclerosis population. J Neurol Sci.

[5] Ahmad H, Palmer A, Campbell JA, van der Mei I, Taylor B (2018). Health economic impact of multiple sclerosis in Australia in 2017.

[6] Browne P, Chandraratna D, Angood C, Tremlett H, Baker C, Taylor BV, Thompson AJ (2014). Atlas of multiple sclerosis 2013: a growing global problem with widespread inequity. Neurology.

[7] Australia, M. S. S. o. 2017. "Symptoms." 2019, from https://www.msaustralia.org.au/about-ms/symptoms.

[8] Kurtzke JF, Beebe GW, Norman JE (1979). Epidemiology of multiple sclerosis in U.S. veterans: 1. Race, sex, and geographic distribution. Neurology.

[9] Zuvich RL, McCauley JL, Pericak-Vance MA, Haines JL (2009). Genetics and pathogenesis of multiple sclerosis. Semin Immunol.

[10] Gooch CL, Pracht E, Borenstein AR (2017). The burden of neurological disease in the United States: a summary report and call to action. Ann Neurol.

[11] Oksenberg JR, Baranzini SE, Sawcer S, Hauser SL (2008). The genetics of multiple sclerosis: SNPs to pathways to pathogenesis. Nat Rev Genet.

[12] Willer CJ, Dyment DA, Sadovnick AD, Rothwell PM, Murray TJ, Ebers GC, G. Canadian Collaborative Study (2005). Timing of birth and risk of multiple sclerosis: population based study. BMJ.

[13] Compston A, Coles A (2008). Multiple sclerosis. Lancet.

[14] Trapp BD, Nave KA (2008). Multiple sclerosis: an immune or neurodegenerative disorder?. Annu Rev Neurosci.

[15] Rosati G (2001). The prevalence of multiple sclerosis in the world: an update. Neurol Sci.

[16] Hauser SL, Oksenberg JR (2006). The neurobiology of multiple sclerosis: genes, inflammation, and neurodegeneration. Neuron.

[17] Huijbregts SC, Kalkers NF, de Sonneville LM, de Groot V, Polman CH (2006). Cognitive impairment and decline in different MS subtypes. J Neurol Sci.

[18] Huijbregts SC, Kalkers NF, de Sonneville LM, de Groot V, Reuling IE, Polman CH (2004). Differences in cognitive impairment of relapsing remitting, secondary, and primary progressive MS. Neurology.

[19] Brady ST, Witt AS, Kirkpatrick LL, de Waegh SM, Readhead C, Tu PH, Lee VM (1999). Formation of compact myelin is required for maturation of the axonal cytoskeleton. J Neurosci.

[20] Olerup O, Hillert J (1991). HLA class II-associated genetic susceptibility in multiple sclerosis: a critical evaluation. Tissue Antigens.

[21] Haines JL, Terwedow HA, Burgess K, Pericak-Vance MA, Rimmler JB, Martin ER, Oksenberg JR, Lincoln R, Zhang DY, Banatao DR, Gatto N, Goodkin DE, Hauser SL (1998). Linkage of the MHC to familial multiple sclerosis suggests genetic heterogeneity. The Multiple Sclerosis Genetics Group. Hum Mol Genet.

[22] Oksenberg JR, Baranzini SE (2010). Multiple sclerosis genetics--is the glass half full, or half empty?. Nat Rev Neurol.

[23] Baranzini SE, Wang J, Gibson RA, Galwey N, Naegelin Y, Barkhof F, Radue EW, Lindberg RL, Uitdehaag BM, Johnson MR, Angelakopoulou A, Hall L, Richardson JC, Prinjha RK, Gass A, Geurts JJ, Kragt J, Sombekke M, Vrenken H, Qualley P, Lincoln RR, Gomez R, Caillier SJ, George MF, Mousavi H, Guerrero R, Okuda DT, Cree BA, Green AJ, Waubant E, Goodin DS, Pelletier D, Matthews PM, Hauser SL, Kappos L, Polman CH, Oksenberg JR (2009). Genome-wide association analysis of susceptibility and clinical phenotype in multiple sclerosis. Hum Mol Genet.

[24] Gregory SG, Schmidt S, Seth P, Oksenberg JR, Hart J, Prokop A, Caillier SJ, Ban M, Goris A, Barcellos LF, Lincoln R, McCauley JL, Sawcer SJ, Compston DA, Dubois B, Hauser SL, Garcia-Blanco MA, Pericak-Vance MA, Haines JL, G. Multiple Sclerosis Genetics (2007). Interleukin 7 receptor alpha chain (IL7R) shows allelic and functional association with multiple sclerosis. Nat Genet.

[25] Hafler CDA, Compston A, Sawcer S, Lander ES, Daly MJ, De Jager PL, de Bakker PI, Gabriel SB, Mirel DB, Ivinson AJ, Pericak-Vance MA, Gregory SG, Rioux JD, McCauley JL, Haines JL, Barcellos LF, Cree B, Oksenberg JR, Hauser SL, International Multiple Sclerosis Genetics (2007). Risk alleles for multiple sclerosis identified by a genomewide study. N Engl J Med.

[26] Lundmark F, Duvefelt K, Iacobaeus E, Kockum I, Wallstrom E, Khademi M, Oturai A, Ryder LP, Saarela J, Harbo HF, Celius EG, Salter H, Olsson T, Hillert J (2007). Variation in interleukin 7 receptor alpha chain (IL7R) influences risk of multiple sclerosis. Nat Genet.

[27] Xue HH, Kovanen PE, Pise-Masison CA, Berg M, Radovich MF, Brady JN, Leonard WJ (2002). IL-2 negatively regulates IL-7 receptor alpha chain expression in activated T lymphocytes. Proc Natl Acad Sci U S A.

[28] Lee JS, Chien CB (2004). When sugars guide axons: insights from heparan sulphate proteoglycan mutants. Nat Rev Genet.

[29] Cassaro CM, Dietrich CP (1977). Distribution of sulfated mucopolysaccharides in invertebrates. J Biol Chem.

[30] Nader HB, Chavante SF, dos-Santos EA, Oliveira TW, de-Paiva JF, Jeronimo SM, Medeiros GF, de-Abreu LR, Leite EL, de-Sousa-Filho JF, Castro RA, Toma L, Tersariol IL, Porcionatto MA, Dietrich CP (1999). Heparan sulfates and heparins: similar compounds performing the same functions in vertebrates and invertebrates?. Braz J Med Biol Res.

[31] Habuchi H, Habuchi O, Kimata K (2004). Sulfation pattern in glycosaminoglycan: does it have a code?. Glycoconj J.

[32] Inatani M, Yamaguchi Y (2003). Gene expression of EXT1 and EXT2 during mouse brain development. Brain Res Dev Brain Res.

[33] Jennes I, Zuntini M, Mees K, Palagani A, Pedrini E, De Cock G, Fransen E, Vanden Berghe W, Sangiorgi L, Wuyts W (2012). Identification and functional characterization of the human EXT1 promoter region. Gene.

[34] Holst CR, Bou-Reslan H, Gore BB, Wong K, Grant D, Chalasani S, Carano RA, Frantz GD, Tessier-Lavigne M, Bolon B, French DM, Ashkenazi A (2007). Secreted sulfatases Sulf1 and Sulf2 have overlapping yet essential roles in mouse neonatal survival. PLoS One.

[35] Lin X, Wei G, Shi Z, Dryer L, Esko JD, Wells DE, Matzuk MM (2000). Disruption of gastrulation and heparan sulfate biosynthesis in EXT1-deficient mice. Dev Biol.

[36] Dreyfuss JL, Regatieri CV, Jarrouge TR, Cavalheiro RP, Sampaio LO, Nader HB (2009). Heparan sulfate proteoglycans: structure, protein interactions and cell signaling. An Acad Bras Cienc.

[37] Han CH, Huang YJ, Lu KH, Liu Z, Mills GB, Wei Q, Wang LE (2011). Polymorphisms in the SULF1 gene are associated with early age of onset and survival of ovarian cancer. J Exp Clin Cancer Res.

[38] Sahota AP, Dhoot GK (2009). A novel SULF1 splice variant inhibits Wnt signalling but enhances angiogenesis by opposing SULF1 activity. Exp Cell Res.

[39] Nagamine S, Tamba M, Ishimine H, Araki K, Shiomi K, Okada T, Ohto T, Kunita S, Takahashi S, Wismans RG, van Kuppevelt TH, Masu M, Keino-Masu K (2012). Organ-specific sulfation patterns of heparan sulfate generated by extracellular sulfatases Sulf1 and Sulf2 in mice. J Biol Chem.

[40] Haupt LM, Griffiths LR (2009). Heparan Sulfate Proteoglycans, Tumour Progression and the Cancer Stem Cell Niche. Curr Cancer Ther Rev.

[41] Haupt LM, Murali S, Mun FK, Teplyuk N, Mei LF, Stein GS, van Wijnen AJ, Nurcombe V, Cool SM (2009). The heparan sulfate proteoglycan (HSPG) glypican-3 mediates commitment of MC3T3-E1 cells toward osteogenesis. J Cell Physiol.

[42] Lindahl U, Kusche-Gullberg M, Kjellen L (1998). Regulated diversity of heparan sulfate. J Biol Chem.

[43] Tumova S, Hatch BA, Law DJ, Bame KJ (1999). Basic fibroblast growth factor does not prevent heparan sulphate proteoglycan catabolism in intact cells, but it alters the distribution of the glycosaminoglycan degradation products. Biochem J.

[44] Filmus J, Shi W, Wong ZM, Wong MJ (1995). Identification of a new membrane-bound heparan sulphate proteoglycan. Biochem J.

[45] Malavaki CJ, Theocharis AD, Lamari FN, Kanakis I, Tsegenidis T, Tzanakakis GN, Karamanos NK (2011). Heparan sulfate: biological significance, tools for biochemical analysis and structural characterization. Biomed Chromatogr.

[46] Tkachenko E, Rhodes JM, Simons M (2005). Syndecans: new kids on the signaling block. Circ Res.

[47] David G (1993). Integral membrane heparan sulfate proteoglycans. FASEB J.

[48] Bernfield M, Gotte M, Park PW, Reizes O, Fitzgerald ML, Lincecum J, Zako M (1999). Functions of cell surface heparan sulfate proteoglycans. Annu Rev Biochem.

[49] Ling L, Murali S, Dombrowski C, Haupt LM, Stein GS, van Wijnen AJ, Nurcombe V, Cool SM (2006). Sulfated glycosaminoglycans mediate the effects of FGF2 on the osteogenic potential of rat calvarial osteoprogenitor cells. J Cell Physiol.

[50] Filmus J, Capurro M, Rast J (2008). Glypicans. Genome Biol.

[51] Lorentzen AR, Melum E, Ellinghaus E, Smestad C, Mero IL, Aarseth JH, Myhr KM, Celius EG, Lie BA, Karlsen TH, Franke A, Harbo HF (2010). Association to the Glypican-5 gene in multiple sclerosis. J Neuroimmunol.

[52] Comabella M, Craig DW, Camina-Tato M, Morcillo C, Lopez C, Navarro A, Rio J, Biomarker MSSG, Montalban X, Martin R (2008). Identification of a novel risk locus for multiple sclerosis at 13q31.3 by a pooled genome-wide scan of 500,000 single nucleotide polymorphisms. PLoS One.

[53] Cavanillas ML, Fernandez O, Comabella M, Alcina A, Fedetz M, Izquierdo G, Lucas M, Cenit MC, Arroyo R, Vandenbroeck K, Alloza I, Garcia-Barcina M, Antiguedad A, Leyva L, Gomez CL, Olascoaga J, Otaegui D, Blanco Y, Saiz A, Montalban X, Matesanz F, Urcelay E (2011). Replication of top markers of a genome-wide association study in multiple sclerosis in Spain. Genes Immun.

[54] Tajouri L, Mellick AS, Tourtellotte A, Nagra RM, Griffiths LR (2005). An examination of MS candidate genes identified as differentially regulated in multiple sclerosis plaque tissue, using absolute and comparative real-time Q-PCR analysis. Brain Res Brain Res Protoc.

[55] van Horssen J, Bo L, Dijkstra CD, de Vries HE (2006). Extensive extracellular matrix depositions in active multiple sclerosis lesions. Neurobiol Dis.

[56] Okolicsanyi RK, van Wijnen AJ, Cool SM, Stein GS, Griffiths LR, Haupt LM. "Heparan sulfate proteoglycans and human breast cancer epithelial cell tumorigenicity." J Cell Biochem. 2014;115(5):967–76. 10.1002/jcb.24746 PMC4225069 24357546

[57] Cenit MD, Blanco-Kelly F, de las Heras V, Bartolome M, de la Concha EG, Urcelay E, Arroyo R, Martinez A (2009). Glypican 5 is an interferon-beta response gene: a replication study. Mult Scler.

[58] Karlsen TH, Franke A, Melum E, Kaser A, Hov JR, Balschun T, Lie BA, Bergquist A, Schramm C, Weismuller TJ, Gotthardt D, Rust C, Philipp EE, Fritz T, Henckaerts L, Weersma RK, Stokkers P, Ponsioen CY, Wijmenga C, Sterneck M, Nothnagel M, Hampe J, Teufel A, Runz H, Rosenstiel P, Stiehl A, Vermeire S, Beuers U, Manns MP, Schrumpf E, Boberg KM, Schreiber S (2010). Genome-wide association analysis in primary sclerosing cholangitis. Gastroenterology.

[59] Busse M, Kusche-Gullberg M (2003). In vitro polymerization of heparan sulfate backbone by the EXT proteins. J Biol Chem.

[60] Kim BT, Kitagawa H, Tanaka J, Tamura J, Sugahara K (2003). In vitro heparan sulfate polymerization: crucial roles of core protein moieties of primer substrates in addition to the EXT1-EXT2 interaction. J Biol Chem.

[61] Busse-Wicher M, Wicher KB, Kusche-Gullberg M (2014). The exostosin family: proteins with many functions. Matrix Biol.

[62] Dutta R, Trapp BD (2014). Relapsing and progressive forms of multiple sclerosis: insights from pathology. Curr Opin Neurol.

[63] Goldenberg MM (2012). Multiple sclerosis review. P T.

[64] van Horssen J, Dijkstra CD, de Vries HE (2007). The extracellular matrix in multiple sclerosis pathology. J Neurochem.

[65] Titelbaum DS, Degenhardt A, Kinkel RP (2005). Anti-tumor necrosis factor alpha-associated multiple sclerosis. AJNR Am J Neuroradiol.

[66] Poliseno L, Salmena L, Zhang J, Carver B, Haveman WJ, Pandolfi PP (2010). A coding-independent function of gene and pseudogene mRNAs regulates tumour biology. Nature.

[67] Lendorf ME, Manon-Jensen T, Kronqvist P, Multhaupt HA, Couchman JR (2011). Syndecan-1 and syndecan-4 are independent indicators in breast carcinoma. J Histochem Cytochem.

[68] Okolicsanyi RK, van Wijnen AJ, Cool SM, Stein GS, Griffiths LR, Haupt LM (2014). Heparan sulfate proteoglycans and human breast cancer epithelial cell tumorigenicity. J Cell Biochem.

[69] Celie JW, Beelen RH, van den Born J (2009). Heparan sulfate proteoglycans in extravasation: assisting leukocyte guidance. Front Biosci (Landmark Ed).

[70] Capurro MI, Xiang YY, Lobe C, Filmus J (2005). Glypican-3 promotes the growth of hepatocellular carcinoma by stimulating canonical Wnt signaling. Cancer Res.

[71] Veugelers M, De Cat B, Ceulemans H, Bruystens AM, Coomans C, Durr J, Vermeesch J, Marynen P, David G (1999). Glypican-6, a new member of the glypican family of cell surface heparan sulfate proteoglycans. Jbb Biol Chem.

[72] Li Y, Sheu CC, Ye Y, de Andrade M, Wang L, Chang SC, Aubry MC, Aakre JA, Allen MS, Chen F, Cunningham JM, Deschamps C, Jiang R, Lin J, Marks RS, Pankratz VS, Su L, Li Y, Sun Z, Tang H, Vasmatzis G, Harris CC, Spitz MR, Jen J, Wang R, Zhang ZF, Christiani DC, Wu X, Yang P (2010). Genetic variants and risk of lung cancer in never smokers: a genome-wide association study. Lancet Oncol.

[73] Handel AE, Ramagopalan SV (2010). GPC5 and lung cancer in multiple sclerosis. Lancet Oncol.

[74] Byun E, Caillier SJ, Montalban X, Villoslada P, Fernandez O, Brassat D, Comabella M, Wang J, Barcellos LF, Baranzini SE, Oksenberg JR (2008). Genome-wide pharmacogenomic analysis of the response to interferon beta therapy in multiple sclerosis. Arch Neurol.

[75] Saunders S, Paine-Saunders S, Lander AD (1997). Expression of the cell surface proteoglycan glypican-5 is developmentally regulated in kidney, limb, and brain. Dev Biol.

[76] Van Vactor D, Wall DP, Johnson KG (2006). Heparan sulfate proteoglycans and the emergence of neuronal connectivity. Curr Opin Neurobiol.

[77] Chernousov MA, Rothblum K, Stahl RC, Evans A, Prentiss L, Carey DJ (2006). Glypican-1 and alpha4(V) collagen are required for Schwann cell myelination. J Neurosci.

[78] Fransson LA (2003). Glypicans. Int J Biochem Cell Biol.

