# Supplementary material for: An investigation of genetic polymorphisms in heparan sulfate proteoglycan core proteins and key modification enzymes in an Australian Caucasian multiple sclerosis population
Source: Hum Genomics. 2020 May 12;14:18. doi: 10.1186/s40246-020-00264-6 (PMC7218574; doi:10.1186/s40246-020-00264-6)
Supplement: Supplementary file 1 — Additional file 1: Table S1. Male results by disease state for GPC6, rs17267815. [file 40246_2020_264_MOESM1_ESM.docx]

|  | **GPC6** | **rs17267815** |  |  |  |  |  |  |  |
| --- | --- | --- | --- | --- | --- | --- | --- | --- | --- |
| **Polymorphism** | **Genotypes** |  |  |  |  | **Alleles** |  |  |  |
| **Group** | **AA (%)** | **AG (%)** | **GG (%)** | ***P*** |  | **A(%)** | **G (%)** | ***P*** | **OR (95% CI)** |
| **Male MS Case** |  |  |  |  |  |  |  |  |  |
| **PP (n = 19)** | 3 (15.8) | 9 (47.4) | 7 (36.8) | 0.9533 |  | 15 (39.5) | 23 (60.5) | 0.7826 | 0.89 |
| **SP (n = 11)** | 3 (27.3) | 6 (54.6) | 2 (18.2) | 0.3383 |  | 12 (54.6) | 10 (45.4) | 0.1407 | 0.48 |
| **RR (n = 15)** | 8 (53.3) | 5 (33.3) | 2 (13.3) | **0.0134** |  | 21 (70) | 9 (30) | **0.0024** | 0.25 |
| **Total Control (n = 34)** | 5 (14.7) | 15 (44.1) | 14 (41.2) | - |  | 25 (36.8) | 43 (63.2) | - | - |
| **HapMap CEU (%)** | 26.5 | 54 | 19.5 |  |  | 53.5 | 46.5 |  |  |
